# Antigen-free control wells in an ELISA set-up for the determination of autoantibodies against G protein-coupled receptors—a requisite for correct data evaluation

**DOI:** 10.1007/s00216-018-1172-x

**Published:** 2018-06-11

**Authors:** Annekathrin Haberland, Johannes Müller, Gerd Wallukat, Katrin Wenzel

**Affiliations:** Berlin Cures GmbH, Robert-Rössle-Str. 10, 13125 Berlin, Germany

**Keywords:** Autoantibodies against G protein-coupled receptors, Assay, ELISA, Non-coated well, Solid-phase assay

## Abstract

First functional acting autoantibodies against G protein-coupled receptors such as the beta2-adrenoceptor in e.g. asthmatic patients have already been discovered in the early 1980s of the last century using assays that show their functional activity. Today, almost 40 years later, the measurement of such autoantibodies is still a challenge. Bioassays able to show the functional activity of such autoantibodies against G protein-coupled receptors are still the ne plus ultra for their detection and also classification when additionally exploiting specific receptor blockers for the neutralisation of the effect. Bioassays based on living cells make specific demands on the laboratories and are, therefore not suitable for every routine laboratory. Routine diagnostics, therefore, ideally requires different assays based on e.g. solid-phase technology, such as enzyme-linked immunosorbent assay (ELISA) technology. Here, endeavours are going on, using either the exact epitopes of such autoantibodies, if known, for trapping the autoantibodies, or the complete receptor in biological or artificial membranes that are immobilised onto a plastic carrier (ELISA principle). Here, we question and discuss the outcome of such tests, especially, if no controls such as the non-coated plastic carrier or the corresponding receptor-free membrane coat is offered as control in parallel, in light of the manifold experiences already collected with even non-agonistic acting autoantibodies by Güven et al. (J Immunol Methods 403:26–36, 2014).

## Introduction

An interesting discussion about proper controls in enzyme-linked immunosorbent assay (ELISA) technology has been brought up in the ResearchGate community by Jaya Gosala [1], catching our attention. The researcher Jaya Gosala has been asking, what it tells someone if an antigen-free (non-coated) well, which has been used as control, shows a measuring signal (high background). Of course, a lot of answers appeared. Some stating that this is not the correct control, and/or the blocking buffers have to be improved. Some of the answers appear to be superficial. They did not reach to the root of the problem according to our feeling. We have been wondering about the same problem for some time, while testing solid-phase assays for the detection of functional acting autoantibodies against G protein-coupled receptors, and did not come to an answer about its meaning yet.

Therefore, we would like to not only pick up this discussion but also widen it. What does it tell you if an antigen-free control plate shows a somewhat similar pattern (outcome, result) compared to the antigen-coated measuring ELISA plate—even marketed ELISA plates?

With a ready to use ELISA plate, the buffers and blocking solutions are given—the extinctions (ODs) can be compared to standard curves. In cases, when the ELISA is, however, released for research and development purposes only, the standard curve material does not have to be exactly identical to the analyte, which makes the interpretation of the results more complicated and uncertain. Differences might be possible as it was seen by Wenzel et al. [2] while comparing the outcome of an ELISA using autoantibodies of animal and human origin. While the animal-originated material worked just fine, this was not the case with human material. But, one could even neglect the standard material and just compare different samples of supposably low or high analyte amounts. You do not even need to look at the standard curve at all in such cases and just compare the ODs of the samples.

The crucial question now is, what actually happens if an analyte-free ELISA control plate, which of course has to be activated first with coating buffer comparable to the specific antigen-coated plate, will produce a similar pattern of ODs? What does this tell you? Was the coating not complete? (which is definitely not the case, since the ELISA works just fine, when antibodies, raised against the immunogen peptide in an animal, will be trapped on this plate [2]). Or, is the analyte just a charged molecule, which will stick more or less to whatever ground (activated plastic and/or antigen)—this way giving completely fake or/and overlapping results? And, is it even possible that the coating reduces the non-specific binding without introducing specificity?

Would that explain why in some cases, as reported from autoantibodies against G protein-coupled receptors, researchers find for certain patients/diseases clusters of autoantibodies of this class while using solid-phase ELISA technology?

In especially such cases, it should be tested if the samples also bind onto antigen-free activated plastic plates, ideally exploiting the same plastic matrix, dilution, dilution and washing buffers, and blocking solutions. If not at hand, just use conventional ELISA buffers, they are often used and not completely pivotal. If now the result with a non-coated antigen-free control plate is somewhat similar to that with the specific antigen-coated plate, then this would be enough answer anyway. If not, the antigen-coated plate might be specific but does not have to be.

Here, we simulated such a constellation using a target-peptide ELISA developed and published by Nagatomo et al. [3] for the detection of autoantibodies against the beta1-adrenoceptor.

## Material and methods

### Material

Material of human origin for the autoantibody preparation was obtained from eluate material from the regeneration of an IgG immunoadsorption column from dilated cardiomyopathy patients treated at the Deutsches Herzzentrum Berlin, Berlin, Germany. Donors signed an informed consent form [4]. The coating peptide HWWRAESDEARRCYNDPKCCDFVTNR (corresponding to the second extracellular loop of the beta1-adrenoceptor) was synthesised by Biosyntan, Gesellschaft für bioorganische Synthese GmbH, Berlin, Germany. AffiniPure goat anti-human IgG (H+L)-POD (cat. no. 109-035-003) was purchased from Dianova, Hamburg, Germany.

### IgG preparation

IgG fractions of the samples were prepared using stepwise ammonium sulphate precipitation, as described in detail by Wenzel et al. [2].

### Enzyme-linked immunosorbent assay technology

Specific wells were coated with 2.5 μg target-peptide/well (target-peptide corresponding to the second extracellular loop of the beta1-adrenoceptor, HWWRAESDEARRCYNDPKCCDFVTNR, for trapping autoantibodies directed at this second extracellular loop) freshly dissolved in 50 μL/well 0.1 M carbonate coating buffer overnight at 4 °C according to Nagatomo et al. [3]. Control wells (non-coated) were incubated with the peptide-free coating buffer only.

After removing the coating buffers and washing (3 × 200 μL, washing buffer: 20 mmol/L Tris-HCl, 150 mmol/L NaCl, 4 mmol/L KCl, and 0.1% Tween 20, pH 7.2), the wells were blocked using (*a*–*c*) 1% bovine serum albumin (BSA) and 0.05% Tween 20 in phosphate buffered saline (PBS) or (*d*–*f*) PBS containing 3% skim milk, 0.1% Tween 20, and 0.01% merthiolate (PMT) according to Nagatomo et al. [3]. The plates were incubated at 22 °C for 1 h, the blocking solutions were removed, the plates were washed again, and the samples were applied in (*a*–*c*) conventional dilution buffer consisting of 20 mmol/L Tris-HCl, 150 mmol/L NaCl, 4 mmol/L KCl, and 0.05% Tween 20, pH 7.2 (TVP) or (*d*–*f*) the PMT buffer again and were incubated at 22 °C for 2 h. After decanting the samples and washing, the wells were incubated with the secondary anti-human IgG (H+L)-POD antibody diluted 1:10,000 in 1% BSA/PBS (*a*–*c*) and PMT (*d*–*f*) and after washing the tetramethylbenzidine/H_2_O_2_ detection at 450 nm (reference filter 620 nm) followed, using an Anthos HTII plate reader.

## Results

**Fig. 1:**
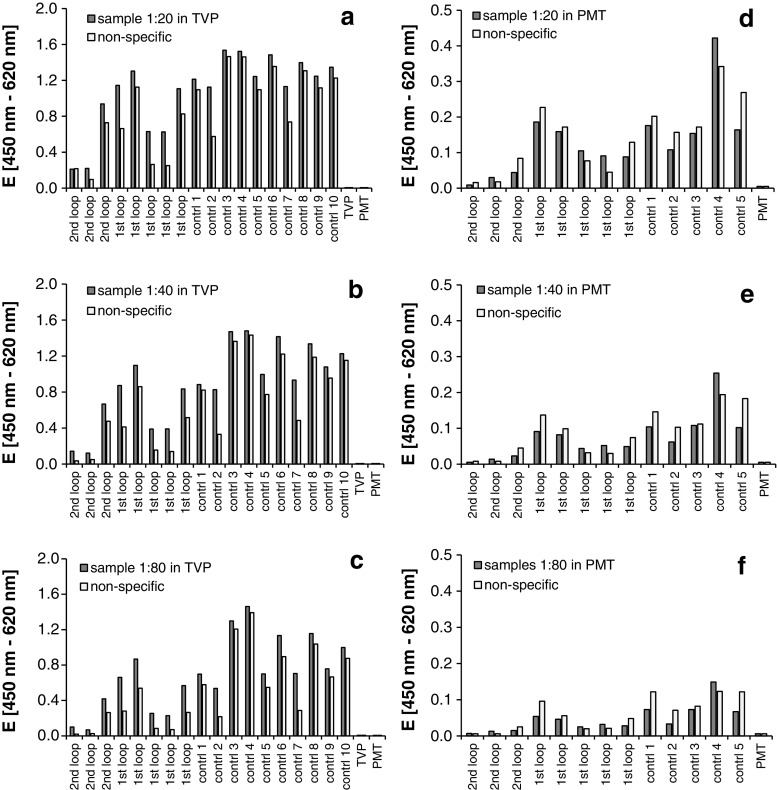
Comparison of the binding of different IgG samples onto specific (target-peptide-antigen-coated) and non-specific (non-coated, coating buffer activated) 96-well ELISA plates. Specific wells (dark grey columns) were coated with 2.5 μg target-peptide/well (target-peptide corresponding to the second extracellular loop of the beta1-adrenoceptor, HWWRAESDEARRCYNDPKCCDFVTNR, for trapping autoantibodies directed at this second extracellular loop) freshly dissolved in 0.1 M carbonate coating buffer overnight at 4 °C according to Nagatomo et al. [3]. Control wells (non-coated, activated wells) were incubated with the peptide-free coating buffer only (light grey columns). IgG samples from healthy donors (“control no. *X*”) and from carriers of beta1-AAbs specific for the first extracellular loop (sample control “1st loop”) and the second extracellular loop (specific samples “2nd loop”) were applied and the binding of the IgGs onto both plates (target-peptide specified and non-coated activated) was compared. According to the discussion forum about this topic in ResearchGate [1], different blocking/buffer conditions were tested. One, the conventional blocking buffer system consisting of 1% BSA/Tween 20 in PBS and the dilution buffer consisting of 20 mmol/L Tris-HCl, 150 mmol/L NaCl, 4 mmol/L KCl, and 0.05% Tween 20, pH 7.2 (TVP) (**a**–**c**) and a second, published by Nagatomo et al. [3], consisting of PBS containing 3% skim milk, 0.1% Tween 20, and 0.01% merthiolate (PMT) (**d**–**f**). Washing conditions were comparable, at each washing step 3 × 200 μL/well washing buffer: 20 mmol/L Tris-HCl, 150 mmol/L NaCl, 4 mmol/L KCl, and 0.1% Tween 20, pH 7.2. The bound IgGs were detected using anti-human IgG (H+L)-POD, (Dianova, Hamburg, Germany, cat no: 109-035-003) at a dilution of 1:10,000 in 1% BSA/PBS (**a**–**c**) or PMT (**d**–**f**) and the TMB/H_2_O_2_ detection system at 450 nm (reference filter 620 nm)

Comparing the OD of the different samples obtained specifically from the peptide-coated wells compared with the non-coated activated wells revealed that at the conventional buffer set-up (Fig. 1a–c), the specific binding was higher than the non-specific binding. Some small differences in the extent of the match between specific and non-specific were seen among the single samples but did not correlate to the sample origin: control (control and autoantibodies specific for first loop) or specific patient (autoantibodies specific for the second loop). This did not change when different sample dilutions (1:20, 1:40, or 1:80) were investigated.

With the buffer set-up published by Nagatomo et al. [3] (Fig. 1d–f), the ODs were smaller in general, but the non-specific binding was mostly equal or in some cases even higher than the so-called “specific” plate. There was no correlation to the sample origin.

## Discussion

This non-specific binding should actually not happen, and the blocking buffers and the whole set-up should have been optimised avoiding such non-specific binding—but, also with the activated non-coated plastic plate? Did everybody check this? As demonstrated with this given example (Fig. 1), this non-specific binding at the non-coated activated plastic plate happens when using very conventional ELISA protocols, as also a protocol published by Nagatomo et al. [3] specifically developed and exploited for the detection of antibodies against the beta1-adrenoceptors.

One might argue that we applied IgG fractions, which might have already a different charge compared to native IgGs in fresh serum. This might be true, but we have already done very many tests even comparing the specific and non-specific binding of fresh serum and the corresponding IgG fractions (data not shown). The results are not much different.

If we now would not have known the sample specifics, we would have attributed a high OD to more autoantibodies (analyte) in the sample bound to the antigen, and low extinction to a lot less autoantibodies bound to the trapping antigen, when only looking at the values generated at the “specific” plate. But, looking at the specific plate is the usual way, a solid-phase ELISA is run and evaluated. One is only looking at the values from the coated plate. A control would be a supposable analyte-free sample (e.g. serum from a healthy person) and/or the dilution buffer only. Everything else stays the same, which includes the coated plate.

The surprising fact that the non-coated, but activated, plate gives a very similar pattern is also excellently investigated in detail and taken together for especially sera from patients suffering from autoimmune diseases by Güven et al. in 2014 [5]. These authors observed that “elevated IgG, elevated IgA, elevated CRP or a combination of these could be observed in all the serum samples with non-specific binding” *…* and, they “speculated that non-specific binding could be a general trait of inflammation/infection”. They also figured out that “non-specific IgG deposition could be found in a number of sera with recent or ongoing bacterial infections”, this way faking “specific” effects.

Terato et al. [6] also described this phenomenon, while especially focusing on sera from autoimmune diseases and developing a special buffer, which was able to reduce this existing problem. These authors demand that in order to “assay antibodies in human sera, it is indispensable to eliminate false positive and negative reactions by using an appropriate buffer system, and to include antigen non-coated blank wells to determine BG (background) noise reactions of individual samples”.

Comparable to our example (Fig. 1), Terato et al. also cite a paper that in “some instances, OD values of control wells can be as high as the values in antigen-coated wells regardless of the antigen”.

If conventional ELISA buffers are applied, should we calculate the difference: coated minus (non-coated) well? Would that be closer to the truth? Or would that add additional nonsense?

As already done by Güven et al. [5] and Terato et al. [6], we would also, therefore, like to invite the autoantibody research community to enlarge the panel of control experiments about the non-specific non-coated plate or a proper control plate, if sophisticated membranes for detection of autoantibodies to membrane receptors are used and to see and report what happens with the results. Just check it out and test it at your set-up.

Also, Güven et al. [5] recommend to acknowledge the existence of this non-specific binding stating that “non-specific binding is thus a significant problem and should be addressed in all routine laboratories measuring human (auto)antibodies”.

The full extent of the problem was recently described in a national laboratory journal in Germany, where end users (patient with support of her physician) carried out and presented intra- and interlaboratory tests in autoimmune diagnostics. The results were partially simply unusable to shocking [7]. Under the headlines “Ist Diagnostik Glücksache” (“Is diagnostics a matter of luck”) and “Diagnostika außer Kontrolle” (“Diagnostics out of control“) by K. Hollricher, the current reality is presented.

Since our experiences accumulate around the autoantibodies against G protein-coupled receptors, we have an interest in learning more about those.

While including antigen-free coating buffer activated control wells into the experimental set-up, we believe the research community can come closer to the truth. We are looking forward to receive not only discussion contributions to this question and problem but also results and experiences from different labs about similar constellations when using solid-phase ELISA technology (including marketed ELISA) for the detection of autoantibodies against G protein-coupled receptors. Such essential controls “are frequently omitted in plates made by investigators conducting basic research, and are not even included in commercially prepared plates” [6].

## References

[1] ELISA negative control which is without coating giving high... In: ResearchGate. https://www.researchgate.net/post/ELISA_negative_control_which_is_without_coating_giving_high_background_not_explaining_the_logic. Accessed 12 Feb 2018.

[2] Wenzel K, Schulze-Rothe S, Müller J, Wallukat G, Haberland A (2018). Difference between beta1-adrenoceptor autoantibodies of human and animal origin—limitations detecting beta1-adrenoceptor autoantibodies using peptide based ELISA technology. PLoS One.

[3] Nagatomo Y, McNamara DM, Alexis JD, Cooper LT, Dec GW, Pauly DF, Sheppard R, Starling RC, Tang WHW, IMAC-2 Investigators (2017). Myocardial recovery in patients with systolic heart failure and autoantibodies against β1-adrenergic receptors. J Am Coll Cardiol.

[4] Müller J, Wallukat G, Dandel M, Bieda H, Brandes K, Spiegelsberger S, Nissen E, Kunze R, Hetzer R (2000). Immunoglobulin adsorption in patients with idiopathic dilated cardiomyopathy. Circulation.

[5] Güven E, Duus K, Lydolph MC, Jørgensen CS, Laursen I, Houen G (2014). Non-specific binding in solid phase immunoassays for autoantibodies correlates with inflammation markers. J Immunol Methods.

[6] Terato K, Do CT, Cutler D, Waritani T, Shionoya H (2014). Preventing intense false positive and negative reactions attributed to the principle of ELISA to re-investigate antibody studies in autoimmune diseases. J Immunol Methods.

[7] Laborjournal 2018-01/03. http://www.laborjournal-archiv.de/epaper/LJ_18_03/16/. Accessed 2 May 2018.

